# Selective Segmentation for Global Optimization of Depth Estimation in Complex Scenes

**DOI:** 10.1155/2013/868674

**Published:** 2013-05-14

**Authors:** Sheng Liu, Haiqiang Jin, Xiaojun Mao, Binbin Zhai, Ye Zhan, Xiaofei Feng

**Affiliations:** ^1^College of Computer Science & Technology, Zhejiang University of Technology, Hangzhou 310023, China; ^2^Key Laboratory of Visual Media Intelligent Processing Technology of Zhejiang Province, Hangzhou 310023, China; ^3^School of Accounting, Zhejiang University of Finance and Economics, Hangzhou 310018, China; ^4^College of Computer and Information Engineering, Zhejiang Gongshang University, Hangzhou 310018, China

## Abstract

This paper proposes a segmentation-based global optimization method for depth estimation. Firstly, for obtaining accurate matching cost, the original local stereo matching approach based on self-adapting matching window is integrated with two matching cost optimization strategies aiming at handling both borders and occlusion regions. Secondly, we employ a comprehensive smooth term to satisfy diverse smoothness request in real scene. Thirdly, a selective segmentation term is used for enforcing the plane trend constraints selectively on the corresponding segments to further improve the accuracy of depth results from object level. Experiments on the Middlebury image pairs show that the proposed global optimization approach is considerably competitive with other state-of-the-art matching approaches.

## 1. Introduction 

Depth estimation from a pair of rectified stereo images is always a challenging research field in vision analysis [1, 2]. The local stereo matching methods often generate outliers in weakly textured areas, discontinuous boundaries, and occlusion areas. Consequently, the global optimization methods [3–7] are designed for more accurate depth estimating in comparison with local ones. Nevertheless, all of these above-mentioned methods neglected the segmentation information in the optimization framework.

The later global optimization methods only partially incorporated the segmentation information into a pixel-level *MRF* model [8–14]. The segmentation information was merely integrated into unary terms or pairwise terms rather than higher order terms. For instance, Wang and Lim [10] proposed a new segment-based stereo matching approach, which takes segments as graph nodes for constructing an irregular segmentation-based graph. In spite of decreasing the computation complexity immensely and showing object-level feature information clearly, it neglected the depth detail and structure detail within the segment and accordingly resulted in the “Mosaic Effect.” 

For taking full advantage of segmentation information, Kohli et al. [15] proposed a higher order term including complete detail of each segment. The *Robust P* ^*n*^ *Potts model* presented by Kohli was originally designed for segmentation applications, which is based on an assumption that the pixels inside the same segment should be label consistency. The labels are used to identify different objects for image segmentation, other than different disparities for depth estimation. So, the energy function for depth estimation cannot penalize the segment with a linear penalty which takes inconsistency pixel ratio into account. Therefore, Kohli's approach is unable to be applied in depth estimation directly. Xie et al. [16] improved the higher order term proposed by Kohli et al. and applied it to the depth estimation successfully. The improved higher order term enforces impliedly the assumption that all the segments of the input image are regarded as various planes. Nevertheless, this assumption is unreasonable because the surfaces of objects are more likely to be irregular surfaces rather than planes in real scene. 

This paper proposes a segmentation-based global optimization method for the depth estimation. Our approach composed of four energy terms makes the following contributions: unlike those familiar data terms converted from local stereo matching methods directly, our data term combines a self-adapting stereo matching approach and two matching cost optimization strategies aiming at occlusion regions and border of image. Most smoothness terms only enforce a simple smoothness strategy over the whole image, which is obviously unable to satisfy the fact that different regions have varying smoothness requirements in a disparity map. Hence, our smoothness term employs a comprehensive smoothness strategy. We incorporate segmentation information in the form of higher order term and perform a selective planarity operation by enforcing a plane trend or not when facing diverse segments. 

**Figure 1:**
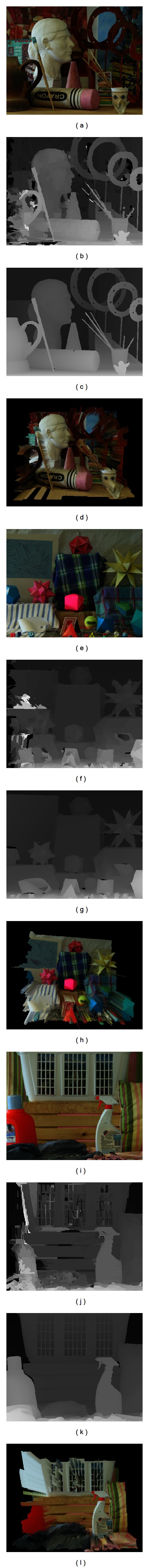
Dense depth maps for the Art, Moebius, and Laundry test sets (from top to bottom). From left to right: the input left images, our final depth maps, ground truth, and three-dimensional reconstructed results. Compared with the ground truth, our results obviously acquire most details of the scene with relatively high accuracy.

Experiment results on the stereo images in Middlebury datasets (Figure 1) have shown that our global optimization method obtains satisfactory depth results and is competitive with the state-of-the-art algorithms. 

## 2. Global Optimization Method for Depth Estimation 

### 2.1. Algorithm Overview

**Figure 2:**
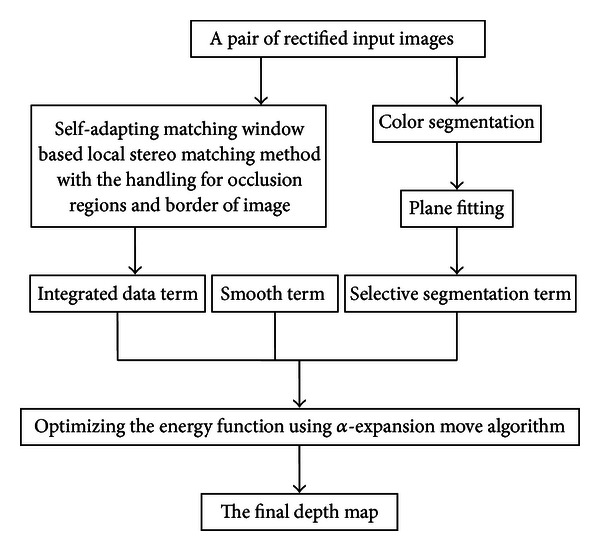
Flow chart of the proposed algorithm.

The input of our algorithm is a pair of rectified stereo images, which are used in improved local stereo matching method based on self-adapting matching windows, color segmentation, and process of constructing smooth term. With the handling of two proposed matching cost optimization strategies, the final matching costs for the pixels are used to not only construct data term but also computer refine map. Both smooth term and segmentation term require the segmentation information produced by [17]. The proposed energy function composed of four energy terms is optimized using **α*-expansion* move algorithm [18]. The whole procedure of our algorithm is illustrated in Figure 2.

### 2.2. Energy Function

In this paper, we presented a segmentation-based global optimization approach composed of *integrated data term, comprehensive smoothness term,* and *selective segmentation term*. To make use of the pixel-level information more adequately, the proposed data term is not only decided by the matching costs from the local stereo matching method based on the improved self-adapting window but also mended the replacement for occlusion regions and evaluation for border of image according to two proposed optimization strategies. Due to comprehensive smoothness strategy, our smooth term is able to satisfy the smoothness requirement more fully. By fusing object-level over-segment information in our global optimization framework, we can richly utilize homogeneous information in the same segment. In addition, the selective planarity operation for segments makes our segmentation term more robust. The global energy function for a unique configuration *f* is as follows:

(1) $$\begin{array}{c} E\left(f\right)=E_{\text{data}}\left(f\right)+E_{\text{smooth}}\left(f\right)+E_{\text{seg}}\left(f\right). \end{array}$$

### 2.3. Data Term Based on Self-Adapting Window

**Figure 3:**
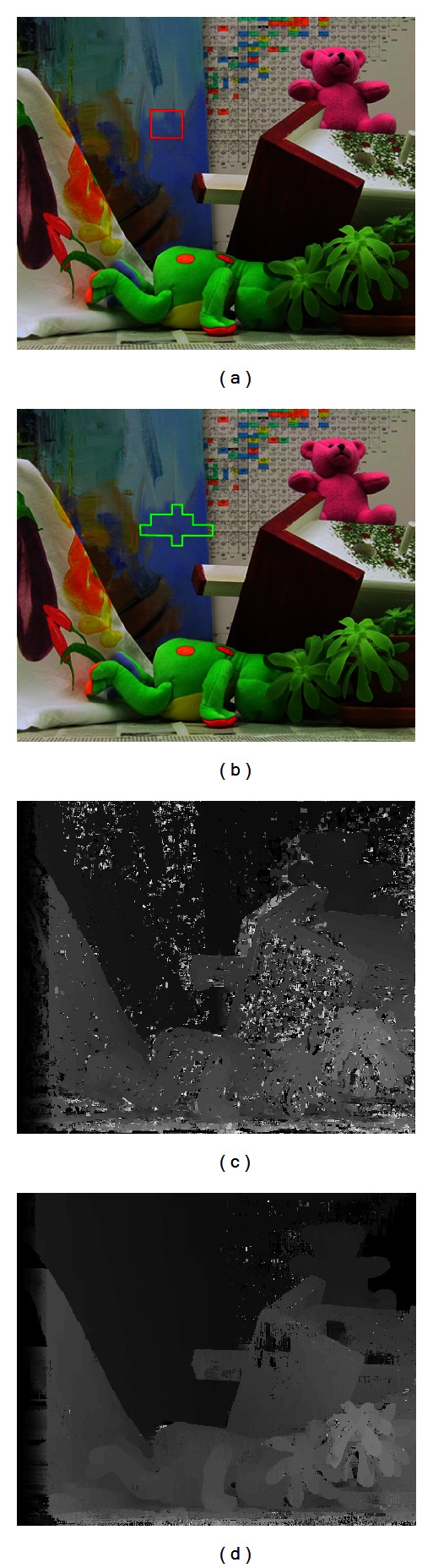
Comparison of local stereo matching methods with fixed matching window and self-adapting matching window for the Teddy (from left to right). Top row: the fixed matching window is marked by red, and the self-adapting matching window is marked by green (from left to right). Bottom row (from left to right): the results by NCC with the fixed matching window, and the results by proposed local stereo matching method with self-adapting matching window. In the NCC case, a mass of obvious outliers occurred in weak-textured regions, discontinuous boundaries, and occlusion areas. The proposed local method has achieved much better results.

In most local stereo matching methods, the fixed matching window is employed for depth estimation. Nevertheless, it is difficult to guarantee that all the pixels in a fixed window are of the same depth. Therefore, there exist amounts of outliers in weak-textured areas, discontinuous boundaries, and occlusion regions shown in Figure 3. In order to improve the accuracy of matching costs for the corresponding depths, the local stereo matching approach based on the self-adapting matching window is adopted for computing the matching costs. 

The local stereo matching approaches with self-adapting matching window are based on the assumption that when pixels with similar intensity within a constrained window have similar disparity, it is necessary to produce an appropriate matching window for each pixel adaptively. In this paper, we mainly refer to the local stereo matching method proposed by Zhang et al. [22] based on self-adapting matching window. Two aspects of improvement are made on the basis of original approach: firstly, a dynamical argument mechanism of minimum window is proposed for more robust correspondence matching. Secondly, we enforce a replacement strategy for occlusion regions and a suboptimum strategy for borders of image. 

Being inspired by five major approaches introduced by Egnal and Wildes [23], we present a replacement strategy to deal with the occlusion regions. Owing to the common assumption that pixels with similar intensity within a neighboring area have similar disparity, the matching costs for occlusion pixels are capable of being replaced with ones for “corresponding” pixels. 

For instance, *d*(*p*) is the disparity for pixel *p* = (*x*_*p*_, *y*_*p*_) in the left input image, and *d*′(*p*′) is the disparity for pixel *p*′ = (*x*_*p*_ − *d*(*p*), *y*_*p*_) in the right image. If *d*(*p*),  *d*′(*p*′) and *d*(*p*′′) satisfy simultaneously the condition that *d*(*p*) > *d*′(*p*′) and *d*′(*p*′) ≤ *d*(*p*′′) where *p*′′ = (*x*_*p*_ − *d*(*p*) + *d*′(*p*′), *y*_*p*_), we would employ a displacement strategy that the matching costs for the pixel *p* in left image are replaced with the one for the pixel *p*′ in right image. 

Neither estimating two disparity maps for left-right consistency check [24, 25] nor applying a simple border extrapolation step, we adopt a suboptimum strategy for the border of image. The corresponding pixel *p*′ will locate outside the right image when (*x*_*p*_ − *d*(*p*)) < 1, which means that the matching cost *C*_*d*_(*p*) cannot be achieved by making use of the corresponding pixels. In this paper, we need the suboptimum label *d* ^∧^,

(2) $$\begin{array}{c} d^{\wedge }=\underset{d\in [d_{\min},d_{\max}],(x_{p}-d)≻0,d\neq d^{*}}{\arg\;\min}C_{d}\left(p\right), \end{array}$$

where d* is the optimal label computed as follows:

(3) $$\begin{array}{c} d^{*}=\underset{d\in [d_{\min},d_{\max}],(x_{p}-d)≻0}{\arg\;\min}C_{d}\left(p\right). \end{array}$$

**Figure 4:**
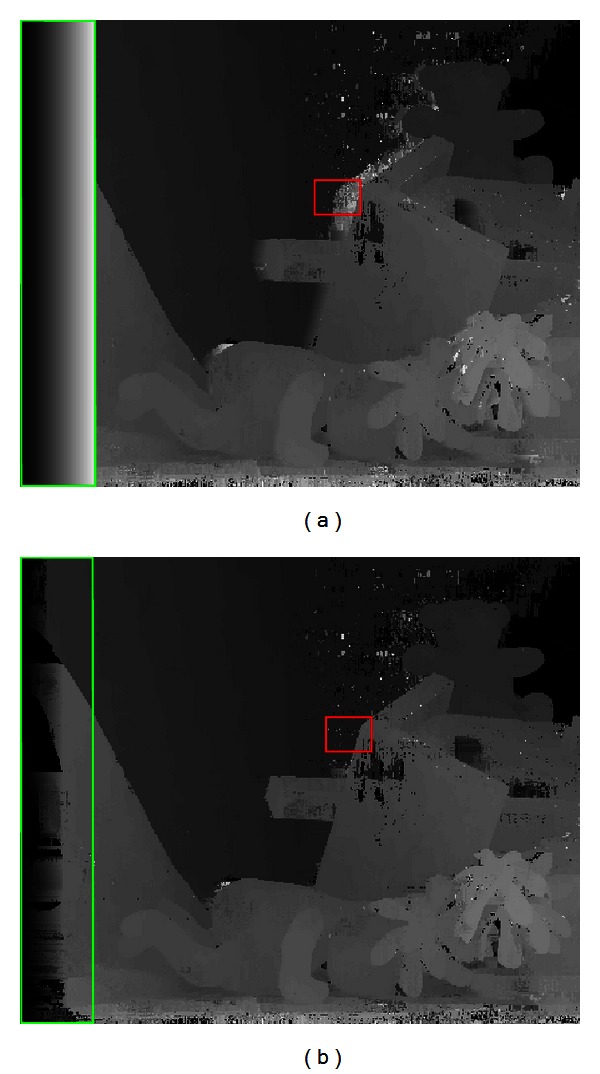
Comparison between the local depth result without occlusion region and border of image (ORBI) handling and the one with ORBI handling for the Teddy (from left to right). Left column: the local depth results without ORBI handling. Right column: the local depth results with ORBI handling. The red frame demonstrates the comparison in occlusion region, while the green frames denote the comparison in border of image. The results show that ORBI handling makes the matching costs for the corresponding depths more reliable.

At last, we use *C*_*d*^∧^_(*p*) as the matching cost for pixel *p* when (*x*_*p*_ − *d*(*p*)) < 1. The improved local results are shown in Figure 4. 

### 2.4. Smooth Term Based on Comprehensive Management

All kinds of smooth terms are presented for smoothing the coarse local results. In this paper, a new comprehensive smooth term is defined based on the similarity of color for dealing with different smoothing requirements on neighborhoods. The proposed smooth term combines the following two smooth terms.

Assume that there is a neighborhood system *N* on the pixel set *P*, *N* ⊂ {(*p*_1_, *p*_2_) | *p*_1_, *p*_2_ ∈ *P*}, Yu et al. [7] performed the consistency of corresponding pixels and their neighbors in their smooth term as follows:

(4) $$\begin{array}{c} E_{\text{smooth}}\left(f\right)=\sum_{(p_{1},p_{2})\in N}\min\left(\left(f\left(p_{1}\right)-f\left(p_{2}\right)\right),k\right), \end{array}$$

where *k* is a constant. 

Kolmogorov and Zabih [3] presented a different smooth term, which considers the color information of corresponding pixels and their neighbors. The smooth term is formulated as follows:

(5) $$\begin{array}{c} E_{\text{smooth}}\left(f\right)=\sum_{(p_{1},p_{2})\in N}V_{p_{1},p_{2}}*T\left(f\left(p_{1}\right)\neq f\left(p_{2}\right)\right), \end{array}$$

where *V*_*p*_1_,*p*_2__ denotes a positive penalty function which imposes disparate penalties according to color differences between pixels. Suppose *R*(*p*), *G*(*p*), and *B*(*p*) are the respective color components of pixel *p* in *RGB* space,

(6) Vp1,p2  ={3λ,if  max⁡⁡(|R(p1)−R(p2)|,|G(p1)−G(p2)|,     |B(p1)−B(p2)|)<ε,λ,otherwise,

where *λ* is a penalty constant, *ε* manages a least color diversity. 

Nevertheless, the smoothness on the boundaries between two adjacent objects will influence the accuracy of the final disparity map. So, we only need to perform the smooth operation in the segments. Compositing the above two kinds of smoothness terms, we propose a new hierarchical smoothness strategy in the identical segment. The new smoothness term is as follows:

(7) Esmooth =∑(p1,p2)∈N,S(p1)=S(p2)Vp1,p2′∗min⁡((f(p1)−f(p2)),k),

where *S*(*p*) is the identification of segment to which the pixel *p* belongs, *V*_*p*_1_,*p*_2__′ denotes a new penalty function which enforces a different penalty on the basis of color differences:

(8) Vp1,p2′={8λif  max⁡⁡(|R(p1)−R(p2)|,|G(p1)−G(p2)|,      |B(p1)−B(p2)|<ε1),2λif  max⁡⁡(|R(p1)−R(p2)|,|G(p1)−G(p2)|,      |B(p1)−B(p2)|<ε2),λif  max⁡⁡(|R(p1)−R(p2)|,|G(p1)−G(p2)|,      |B(p1)−B(p2)|<ε3),σotherwise,  

where *σ* is a penalty constant, *ε*_1_, *ε*_2_, and *ε*_3_ are several color diversities and *ε*_1_ < *ε*_2_ < *ε*_3_. 

The smooth terms perform different smoothness strategies inside the segments according to the diverse color differences of neighborhoods. 

### 2.5. Segmentation Term of Selective Planarity

In this paper, we use the segmentation information to construct the segmentation term for further improving the accuracy of depth estimation. Our segmentation term is different from the higher order term presented by Kohli et al. The higher order term in [15] was originally designed for image segmentation, according to the assumption that the pixels in the same segment should share the same label. However, depth estimation is more likely to satisfy the assumption that the pixels in the same region follow the same distribution such as plane distribution or surface distribution; in other words, the pixels in the same segment could have multiple labels other than only a single label. So, directly making use of Kohli's higher order term for depth estimation is unreasonable.

Obviously, the surface distribution is more representative than the plane distribution because the objects in real scene are more likely composed of irregular surfaces rather than planes. Nevertheless, in this paper the plane distribution is adopted with considering its lower computation complexity and more commonly approximate representativeness. The segments obtained by [17] are further divided into many more subsegments using certain plane distribution. The plane distribution is achieved by plane fitting for the local results. And all the pixels in each subsegment are more likely to share the same label. 

Not all the segments are appropriate to enforce the plane distributions. If the plane distribution is employed roughly in those segments which are unable to be represented by plane, the worse influences on resulting depth map would occur.

**Figure 5:**
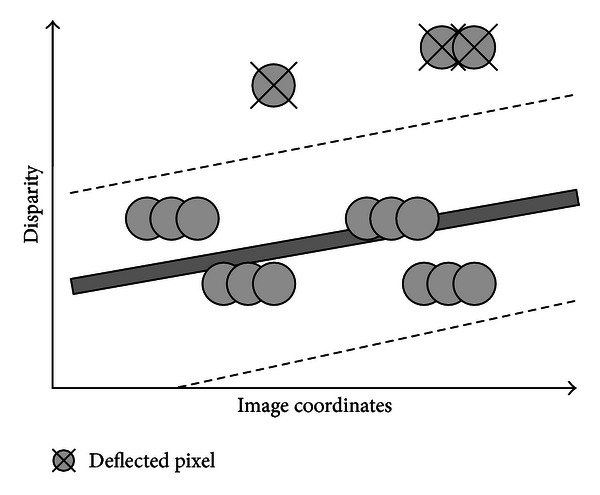
Sketch map for deflected pixels.

In this paper, before performing the plane distributions in the segments, we employ a segment classify procedure for every segment using a proposed plane-judge approach as shown in Figure 5. 

For instance, the pixel *p* is judged as deflected when it meets the condition that |*f*(*p*) − d*(*p*)|>Φ, where d*(*p*) is the disparity value for the pixel *p* after plane fitting using the local depths, and Φ is a constant that controls the planarity quality of segments. *p*_*S*_ is a pixel set for all pixels in the segment *S*, *N*_*d*_(*f*(*p*_*S*_)) denotes the number of deflected pixels in the segment *S*,  *N*(*p*_*S*_) denotes the number of pixels in the segment *S*, and *μ* ∈ (0,1) controls the planarity level of the “planar” segment. If *N*_*d*_(*f*(*p*_*S*_)) > *N*(*p*_*S*_)∗*μ*, we would not construct a homologous segmentation term for the segment *S*. Otherwise, the segmentation term would be constructed using the *Robust P* ^*n*^ *Potts model*. 

**Figure 6:**
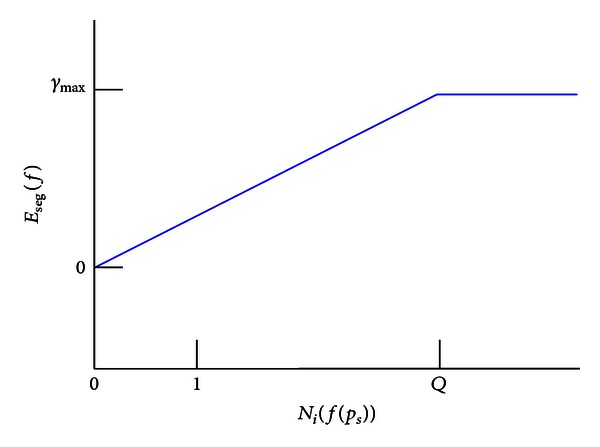
Behavior of the *Robust P* ^*n*^ *Potts function*. The figure shows how the higher order cost of the *Robust p* ^*n*^ *Potts function* changes with the number of pixels in the segment not taking the dominant label.

The segmentation function *E*_seg_(*f*) using the *Robust P* ^*n*^ *Potts model* is defined as

(9) $$\begin{array}{c} E_{\text{seg}}\left(f\right)=\{\begin{array}{cc} N_{i}\left(f\left(p_{S}\right)\right)\frac{1}{Q}\gamma_{\max} & \text{if}\;\;N_{i}\left(f\left(p_{S}\right)\right)\leq Q,\\ \gamma_{\max} & \text{otherwise}, \end{array} \end{array}$$

where *N*_*i*_(*f*(*p*_*S*_)) denotes the number of pixels in the segment *S* not taking the dominant label, *γ*_max⁡_ is the maximum value of label inconsistency cost, and *Q* is the truncation parameter controlling the rigidity of segmentation function. The *Robust P* ^*n*^ *Potts model* proposed by Kohli et al. [15] is shown in Figure 6.

Concrete constructing procedure of segmentation term for each segment is shown in Algorithm 1. 

The segmentation terms enforce the plane trends into the segments which can be represented by plane approximately. 

### 2.6. Energy Minimization Process Based on Graph Cuts

In order to minimize the global energy function by graph cut, all energy terms of this energy function must be submodular according to [26]. In the light of additive principle, if every term in energy function is submodular, the whole global energy function will be submodular. The unary term, such as data term, is always submodular. The pairwise term, namely, smooth term, also is submodular since it satisfies the inequality *E* ^*i*,*j*^(0,0) + *E* ^*i*,*j*^(1,1) ≤ *E* ^*i*,*j*^(0,1) + *E* ^*i*,*j*^(1,0). And from the definition of *Robust p* ^*n*^ *Potts model*, the segmentation term does satisfy the definition of the submodularity on *F* ^*N*^(*N* ≥ 3) [27], if and only if all its projections on two variables are submodular.

According to [26], the segmentation terms can be transformed into sum of pairwise terms:

(10) Eseg=minm0,m1(r0(1−m0)+θdm0∑i∈cdwi(1−ti)+r1m1          +θα(1−m1)∑i∈cwiti−δ).

**Figure 7:**
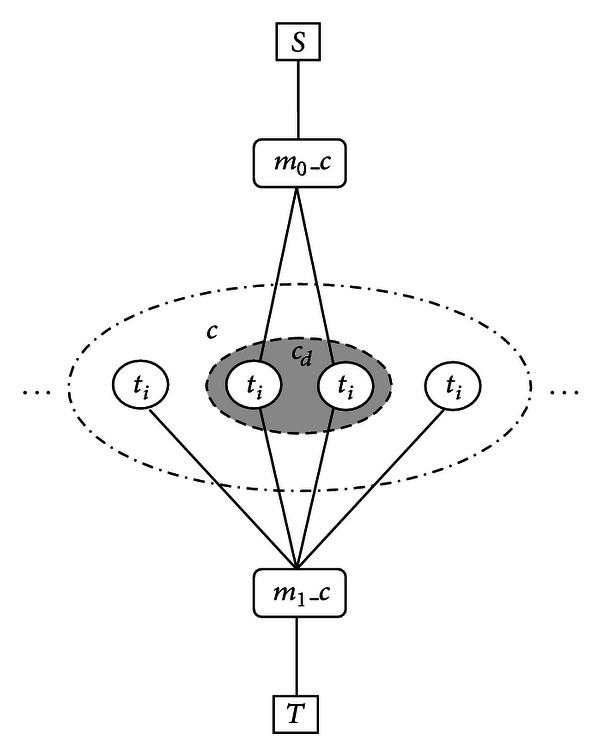
The graph for segmentation terms. *S* is source, *T* is the sink, and *c* represents clique; only two auxiliary nodes, namely, *m*_0_ and *m*_1_ are needed for each clique.

Finally, the global energy function is minimized by utilizing the minimum cut on the graph as shown in Figure 7. The minimum cut can be calculated very efficiently using the **α*-expansion* move algorithm [18]. 

The detailed minimization process is as shown in Algorithm 2.

## 3. Experiment 

Our program is tested by a personal computer with a 2.20 GHz AMD Dual-Core CPU. All data sets are from [28–31]. 

For the Middlebury stereo datasets with four stereo test pairs, that is, Tsukuba, Venus, Teddy, and Cones, Table 1 summarizes the quantitative performance of our method and those of other stereo matching methods, roughly in descending order of overall performance. The comparisons with other approaches show that our global optimization method is fairly competitive with those state-of-the-art approaches. 

**Figure 8:**
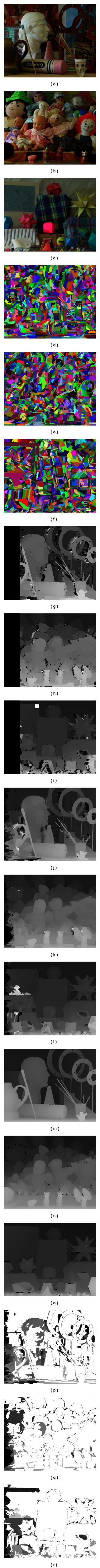
The comparison of final depth maps for the Art, Dolls, and Moebius stereo datasets (from top to bottom). First row: the input left images. Second row: the color segmentation results. Third row: the depth results of regular graph cut. Fourth row: the final depth map of our global optimization method. Fifth row: ground truth. Sixth row: “bad pixel” map of matching results.

For sake of declaring the generality of our global optimization method, abundant other stereo image pairs from Middlebury datasets are adopted for depth estimation.  Figure 8 illustrates that our global optimization method still achieves satisfactory performance on other stereo images. 

## 4. Conclusion and Discussion 

Obviously, the local stereo matching methods based on self-adapting matching window have obtained more outstanding results than fixed matching window based ones. After applying the two proposed matching cost optimization strategies, the local depth results are more accurate in occlusion areas and borders of image. The smooth term makes the surface of segments more close to the real objects. The higher order term, namely, the proposed selective segmentation term, which introduces the plane trend constraint selectively, further enhances the accuracy at object level. In a word, our global optimization method has achieved good performance on Middlebury stereo datasets. 

## Figures and Tables

**Algorithm 1:**
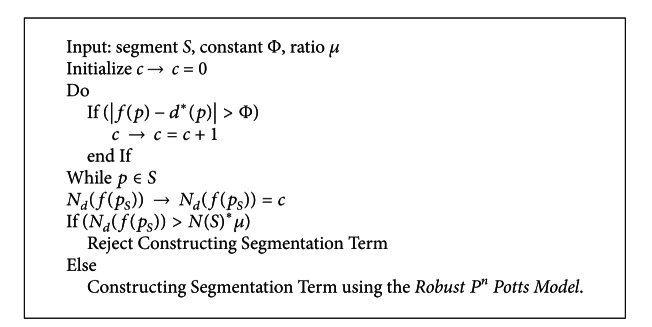
Constructing procedure of segmentation term.

**Algorithm 2:**
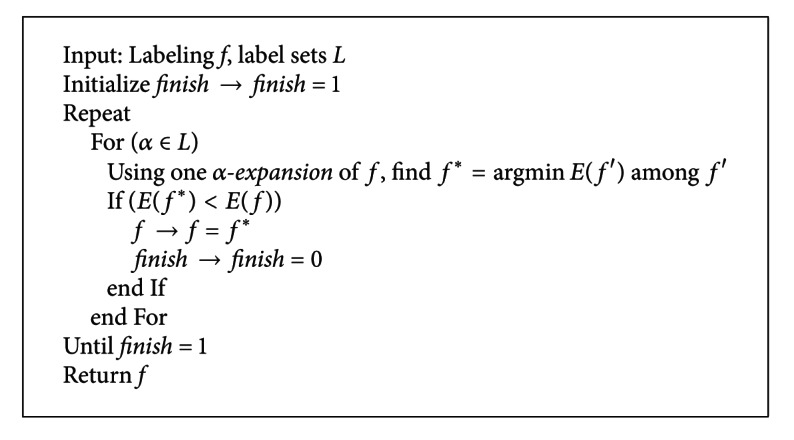
Energy minimization process by graph cut.

**Table 1:** Quantitative evaluation results (bad pixels percentage) of different stereo matching methods for the Tsukuba, Venus, Teddy, and Cones stereo test pairs.

Algorithm	Tsukuba	Venus	Teddy	Cones	Average percent of bad pixels
DCBGrid [19]	5.16	1.23	10.8	9.48	6.67
Our method	2.04	1.58	12.60	12.70	7.23
BioPsyASW [20]	4.91	3.41	14.10	11.30	8.43
CSBP [21]	3.84	2.52	17.30	14.20	9.47
Regular GC	4.43	6.56	39.80	59.00	27.45
